# Carotenoid-Producing Yeasts: Identification and Characteristics of Environmental Isolates with a Valuable Extracellular Enzymatic Activity

**DOI:** 10.3390/microorganisms7120653

**Published:** 2019-12-04

**Authors:** Karolina Chreptowicz, Jolanta Mierzejewska, Jana Tkáčová, Mateusz Młynek, Milan Čertik

**Affiliations:** 1 Drug and Cosmetic Biotechnology, Faculty of Chemistry, Warsaw University of Technology, 00-664 Warsaw, Poland; jmierzejewska@ch.pw.edu.pl (J.M.); mlynekk.m@gmail.com (M.M.); 2 Institute of Biotechnology, Faculty of Chemical and Food Technology, Slovak University of Technology, 812 37 Bratislava, Slovakia; jane.tkacova@gmail.com (J.T.); milan.certik@stuba.sk (M.Č.)

**Keywords:** basidiomycetes, carotenoid pigments, impact of temperature, cellulolytic yeasts

## Abstract

Sixteen cold-adapted reddish-pigmented yeast strains were obtained from environmental samples. According to the PCR-based detection of classical yeast markers combined with phylogenetic studies, the yeasts belong mainly to the genera *Rhodotorula*, *Sporobolomyces* and *Cystobasidium*, all within the subphylum Pucciniomycotina. All strains produced carotenoids within a 0.25–10.33 mg/L range under non-optimized conditions. Noteworthily, among them, representatives of the *Cystobasidium* genus were found; of particular value are the strains *C. laryngis* and *C. psychroaquaticum*, poorly described in the literature to date. Interestingly, carotenoid production with representatives of *Cystobasidium* was improved 1.8- to 10-fold at reduced temperature. As expected, most of the isolated yeasts biosynthesized extracellular lipases, but within them also one proteolytic and four cellulolytic strains were revealed. We succeeded in isolating strain *Cystofilobasidium macerans* WUT145 with extraordinarily high cellulolytic activity at 22°C (66.23 ± 0.15 µmol/mg protein·min) that is described here for the first time. Consequently, a set of yeasts capable of producing both carotenoids and extracellular enzymes was identified. Taking into account those abilities, the strains might be applicable for a development of carotenoids production on an agro-industrial waste, e.g., lignocellulose.

## 1. Introduction

Yeast of the class basidiomycetes prevail among fungi in polar regions [1] and it is estimated that, to date, no more than 5% of the species existing in Nature has been recognised (approx. 50 genera and 250 species). With significant agricultural and medical importance, this group of microorganisms has gained increasing interest among scientists both in terms of their biodiversity and ecological roles, as well as from an economic point of view. It has been found that several basidiomycetous yeasts have a great impact on bio- control of plant diseases, whereas others can break down aromatic compounds and are therefore considered useful in bioremediation [2]. In addition, under particular conditions several fungal species are capable of accumulating lipids in so-called lipid bodies (LB) accounting for up to 65% of their dry biomass. These lipids are of great interest for the cosmetics industry, for several reasons, e.g., emolliency, lubricity; and for the pharmaceutical industry, as delivery agents to provide more homogeneous and efficient application and transport of active agents [3]. Furthermore, some basidiomycetous yeasts produce pigments, mainly carotenoids (also called tetraterpenoids) such as γ- and β-carotene, torulene and torularhodin (*Rhodotorula* spp. and *Sporobolomyces roseus*), or astaxanthin (*Phaffia rhodozyma*) [4,5,6,7,8,9,10]. Natural pigments constitute about 30% of the dyeing substances market [11]. In response to the growing legal restrictions on the use of artificial food additives, under both European and USA legislation, new methods of obtaining natural dyes are widely sought after. Carotenoids are one of the most common classes of pigments, with the global market estimated at nearly $1.4 billion in 2018, and forecasted to reach $1.5–1.8 billion for 2019–2020 [12,13]. They are widely used in the food, pharmaceutical, cosmetic and the nutrition industries. Not just useful as colorants, carotenoids are important molecules in protecting the human and animal body against photo-oxidative damage, including cataracts, an in skin protection and cancer prevention [14,15,16]. Some of them are vitamin A precursors, and thus are marked as high-value nutritional molecules.

Although the microbial production of carotenoids has been extensively studied for a long time, their industrial-scale production remains uneconomical. To reduce the costs of production it would be worthwhile to utilize agricultural waste, e.g., lignocellulose and whey. However, this is no simple task, since these residues are unlikely to be fermented by microorganisms, and need to be pre-treated and hydrolyzed prior to fermentation. This generates additional costs, which could be reduced by applying microorganisms not only capable of producing large amounts of carotenoids, but also of secreting extracellular hydrolase enzymes. Besides, the agricultural wastes are rich in carbon sources, e.g., xylose, cellobiose or lactose, which are not commonly fermented by yeasts. Although several basidiomycetes yeast species were reported to produce hydrolases (e.g., polysaccharases, lipases) [17] or ligninolytic enzymes [18], we still know very little about their application in bioprocesses, e.g., converting organic waste into carotenoids. Among the basidiomycetes the least studied group of yeasts constitute members of the *Cystobasidium* genus. The search for diversity in Nature recently led to the recognition of new yeast species within the *Cystobasidium* genus, such as *Cystobasidium oligophagum, Cystobasidium laryngis, Cystobasidium iriomotense*. They appear to be capable of converting agro industry by-products, and have been proposed as oil or enzyme producers [19,20,21]. Thus, it is of great interest to further explore this field and search for new basidiomycetes yeast and examine their biotechnological potential.

In this study, a set of sixteen reddish-pigmented yeast strains were isolated from plant and food samples, and subsequently characterized with a particular emphasis on their potential for carotenoid, lipid and extracellular enzyme production.

## 2. Materials and Methods

### 2.1. Isolating the Yeast Strains 

The yeast strains were isolated with the procedure previously described in [22] and their molecular characterization is described in the present manuscript. The exception is strain *R. mucilaginosa* WUT10, which has been isolated previously and initially described in [22]. In brief, samples of plant material were spread on standard YPD agar plates (10 g/L yeast extract (Biocorp, Warsaw, Poland), 20 g/L peptone (Biocorp), 20 g/L glucose (Bioshop, Burlington, ON, Canada) and 20 g/L agar (Biocorp)), supplemented with ampicillin and streptomycin providing a final concentration of 100 and 20 mg/L respectively. The plates were then incubated for 2–4 days at 25 °C. The single colonies growing on plates were streaked once again and incubated 2–4 days at 25 °C.

### 2.2. Polymerase Chain Reaction and the Sequencing of rDNA Fragments

Classical yeasts markers (ITS and D1/D2) were used to identify yeast species. The total DNA of the selected strains was extracted and amplified by PCR with a pair of NL4 (5′GGTCCGTGTTTCAAGACGG3′) and ITS1 (5′TCCGTAGGTGAACCTGCGG3′) primers, as described previously [23]. The products of reactions were purified and sequenced (Genomed S.A., Warsaw, Poland). The sequences were deposited in the NCBI GenBank database (Table 1).

### 2.3. Phylogenetic Analysis

Phylogenetic analyses of the obtained sequences were made according to [24] in MEGA-X Program. At first, nucleotide BLAST analysis was done and sequences of reference strains with the sequences of the closest relevant strains (cut-off level: Q_cover_ ≥ 85% and Identity ≥ 90%) were further used in multiple-alignment using MUSCLE program. Aligned sequences were used to construct the phylogenetic tree.

### 2.4. Characterization of Isolated Yeast

Newly isolated yeast strains were checked for the growth on various carbon sources using solid media containing: 6.7 g/L yeast nitrogen base with ammonium sulphate without amino acids (Conda, Madrid, Spain), 20 g/L agar and, respectively, 20 g/L D-glucose, D-xylose (Carl Roth, Karlsruhe, Germany), D-galactose (Carl Roth), lactose (POCH, Gliwice, Poland), D(+)-sucrose (Bioshop), D(+)-cellobiose (Fluka, Buchs, Switzerland) or glycerol (Bioshop) as a single carbon source at 25 °C for 2–4 days. Furthermore, the yeast growth on a rich solid YPD medium was also tested at various temperatures, both low and high, in a range between 4–42 °C.

### 2.5. Screening for Extracellular Enzymes Production 

Isolated yeasts were characterized in terms of their extracellular enzymes production by qualitative plate assays. The selected yeast cultures were grown for 2–3 days at 22 °C on plates containing the following media: the lipase activity was detected on Spirit Blue agar (Becton Dickinson, New Jersey, USA) with 20% (v/v) emulsion olive oil/Tween80, the protease activity was tested on nutrient agar supplemented with skimmed milk and cellulase activity on yeast nitrogen base with ammonium sulphate without amino acids (Conda) with 20 g/L carboxymethylcellulose (CMC) (Sigma Aldrich, St. Louis, MO, USA). The expression of lipase and protease enzymes was confirmed by a zone of clearance around the colony. In case of cellulolytic activity, a positive result was indicated by light halos surrounding isolated colonies after pouring Lugol′s iodine onto the plate.

### 2.6. Determining Cellulolytic Activity in a Plate Assay and Liquid Assay

Selected strains were cultivated overnight in Sabouraud broth at 22 °C and later diluted in a YPDC medium containing 10 g/L yeast extract, 20 g/L peptone, 10 g/L glucose and 10 g/L CMC. The yeast cultures were grown for 4 days at 22 °C and agitated at 240 rpm in Lab Companion SI-600R bench top shaker (Ramsey, MN, USA). Supernatants containing extracellular proteins were obtained by centrifuging the yeast cultures at 10,000 g for 5 min at 4 °C (Sorval Evolution RC Centrifuge, Thermo Scientific, Waltham, MA, USA). The biomass was discarded and the total protein content in the supernatant was determined using the Bradford method.

For CMC degradation plate test, 10 µL of cell-free supernatants containing extracellular proteins were spotted onto the plates containing 2% CMC. Plates were incubated overnight at 22 °C. CMC degradation was indicated by light halos surrounding the spots after pouring Lugol′s iodine onto the plate.

A quantitative liquid enzyme assay was carried out according to the methods of International Union of Pure and Applied Chemistry (IUPAC) [25]. For cellulolytic activity, 0.5 mL of supernatant was incubated with 0.5 mL of 2% CMC in a sodium citrate buffer (0.05 M, pH 4.8) at 22 °C for 17 h. Following the incubation, the reaction was terminated by adding 3.0 mL of DNS reagent (3,5-dinitrosalicylic acid, IUPAC name 2-hydroxy-3,5-dinitrobenzoic acid) to 1 mL of reaction mixture. The probes were incubated in a hot water bath for 5 min, and then cooled in a cold water bath, followed by sample dilutions. The concentration of the reducing sugars obtained were measured spectrophotometrically (Rayleigh VIS-7220G Spectrophotometer, Rayleigh Analytical Instruments, Beijing, China) displaying a standard glucose curve. The enzymatic activity was defined as µmol/mg protein·min.

### 2.7. Carotenoids Production in Liquid Cultures

A loop full of material was used to inoculate 20 mL of YPD medium in 100 mL Erlenmeyer flask. All cultures were grown in triplicate at 15 °C, 20 °C, 22 °C or 25 °C, and agitated at 180 rpm in Lab Companion SI-600R bench top shaker.

Overnight cultures were diluted in 100-mL Erlenmeyer flasks containing 20 mL of production medium [10]: 20 g/L glucose (in selected cases 30 g/L or 40 g/L), 2 g/L (NH_4_)_2_SO_4_ (Avantor, Gliwice, Poland), 1 g/L KH_2_PO_4_, 0.5g/L MgSO_4_ (Avantor), 0.1 g/L CaCl_2_ (Avantor), 2 g/L yeast extract, pH 4.9, to a final concentration of 10^6^ cells/mL, and were cultivated for 4 days at 15 °C, 20 °C, 22 °C or 25 °C and agitated at 180 rpm. All cultures were grown in the Lab Companion SI-600R bench top shaker. All experiments were performed in four parallel cultivations.

### 2.8. Isolation of Total Carotenoids and Lipids from Yeast Biomass

Isolation of total carotenoids and lipids was conducted as described previously [26]. In a few words, cells were harvested via centrifugation at 5000 rpm, for 5 min, RT (Rotina 380 Hettich Zentrifugen, Hettich GmbH & Co., Tuttlingen, Germany), rinsed twice with 0.9% NaCl solution and finally with distilled water. The yeast biomass was frozen, lyophilized and subsequently homogenized with mortar sea sand (Lach-Ner, Neratovice, Czech Republic). Carotenoids, together with lipids, were extracted twice with a chloroform/methanol (2:1 v/v) mixture for 1.5 h at RT. The extracts were washed with distilled water (1.2-fold of total extract volume), stirred vigorously and centrifuged to obtain phase separation. The chloroform layer was filtered through anhydrous Na_2_SO_4_ and evaporated under a vacuum. The carotenoid extracts were re-suspended in n-hexane (99%, HPLC grade) or in mixture n-hexane/chloroform (9:1) where necessary. The total lipid content (L) was determined gravimetrically [26]. Carotenoid distribution was analyzed by high performance liquid chromatography (HPLC).

### 2.9. Dry Biomass Estimation

One mL probes from the 4-day production cultures were used to estimate the dry cell weight (DCW). The cells were harvested by centrifugation in pre-weighed tubes at 13,000 rpm for 3 min, RT (Mikro220 Hettich Zentrifugen), rinsed twice with 0.9% NaCl solution and once with distilled water. The tubes were dried for 2 h at 100 °C and then weighed again. The dry biomass content was calculated using the difference between the weight of the tube with dried biomass and the empty tube. Biomass production was expressed as g/L.

### 2.10. HPLC Analysis of Residual Sugars in Culture Media and Carotenoid Pigments Extracted from Yeast Biomass

Twenty μL of sample was injected into the column (SETREX IEX H+ 300 × 8 mm column, Polymer IEX H form, 8 μm) and the sugars content was analyzed using HPLC (SYKAM chromatograph, Sykam GmbH, Eresing, Germany combined with a RI detector set at 35°C and with thermostatic control at 35°C in order to avoid fluctuations in detector responses). Samples were eluted isocratically using 5 mM H_2_SO_4_ as the mobile phase, at the flow rate 1 mL/min. The residual glucose was identified using the recognised standard; under these conditions glucose eluted at 5.2 min.

Twenty μL of carotenoid extract was injected into the column (BIONACOM Velocity CRT column; 250 × 4.6 mm, 5 μm) and the carotenoid composition was analyzed using HPLC (SYKAM S 1125) equipped with a diode array detector (DAD). The applied solvents were acetone (solvent A) and water (solvent B). The solvent flow rate was 1 mL/min with a gradient of 80% A, 20% B at 0 min, 100% A at 12 min, 100% A at 18min, 80% A and 20% B at 19 min, and maintained for 30 min. The carotenoid pigments were identified using the recognised standards: β-carotene (Sigma Aldrich), γ-carotene (CaroteNature, Münsingen, Switzerland), and torulene (CaroteNature), torularhodin (CaroteNature)).

## 3. Results and Discussion 

### 3.1. Identification of Yeasts Isolated from Plant Samples

**Figure 1:**
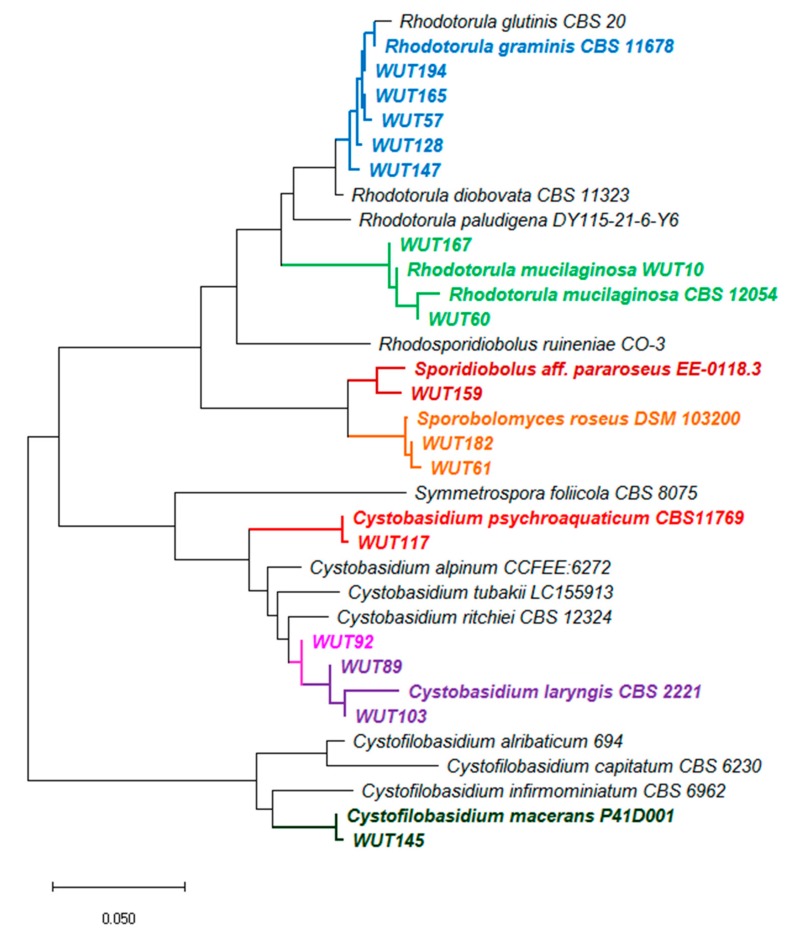
Neighbor-Joining tree showing phylogenetic relationship constructed from the evolutionary distance data for 18S rRNA(partial sequence)-ITS1-5.8S rRNA-ITS2-28S rRNA (partial sequence). The optimal tree with the sum of branch length = 0.93 is shown. The evolutionary distances were computed using the p-distance method and are in the units of the number of base differences per site. This analysis involved 34 nucleotide sequences. Codon positions included were 1st+2nd+3rd+Noncoding. All ambiguous positions were removed for each sequence pair (pairwise deletion option). There were a total of 3349 positions in the final dataset. Evolutionary analyses were conducted in MEGA X [27].

Through screening various plant samples fifteen new red-pink pigmented yeast strains were isolated. Based on sequences of 26S rDNA (D1/D2 domain) and internal transcribed spacer (ITS) region sequences, and using BLAST in the GenBank database combined with phylogenetic analysis, these strains were assigned into five genera (*Rhodotorula, Sporobolomyces, Sporidiobolus, Cystobasidium* and *Cystofilobasidium)* of Basidiomycota division (Table 1, Figure 1). *R. mucilaginosa* WUT10, the previously isolated strain [22], was also included into the present study.

In phylogenetic studies of yeast isolates five main groups were recognised. In the first group, there are five strains directly related to genus *R. graminis* (marked in blue). These strains show slight differences in nucleotide sequences on a common fragment, and between strains WUT194, WUT165 and WUT57 only a six nucleotide (nt) divergence was estimated, whereas between WUT194, WUT128 and WUT147, 5 nt and 7 nt, respectively. The second group comprises of strains related to *R. mucilaginosa* species (marked in green). The highest difference in nucleotide sequence (10 nt) was determined between WUT60 and WUT167, whereas between WUT60 and WUT10 variance in only 6 nt was denoted. Next three strains, WUT159, WUT181 and WUT61, formed individual branches with one direct homologue and were assigned into *Sporidiobolus pararoseus* and *Sporobolomyces roseus* species. In the fourth cluster, there were located strains WUT117, WUT89, WUT103 and WUT92 of *Cystobasidium* genera. In the case of WUT92, phylogenetic analysis did not allow to clearly assign this isolate to the species. It cannot be excluded that WUT92 is a representative of new species, but more complex analysis is required.

### 3.2. Physiological and Biochemical Characteristic of Yeasts 

The initial characterization of the newly isolated strains included: morphological (microscopic observations and determining cells size; Table S1), physiological and biochemical analyses (therein, ability to utilize various carbon sources, range of temperature growth and extracellular enzymes production; Table 2 and Figures S1–S3). Growth observations revealed that all strains metabolize the standard carbon source—glucose, and most of them are also capable of multiplying on a medium containing glycerol (13 strains), cellobiose (13 strains), xylose (13 strains) or maltose (11 strains). Besides, seven isolates, mainly from *Rhodotorula* genus grow well on galactose. As for the range of temperatures, for all 16 strains growth at 25 °C was observed, while at the elevated temperatures it was limited. However, nine strains are able to multiply at 30 °C and one, *R. mucilaginosa* WUT167, at 37 °C. It was observed that the temperature range of growth can vary, even for strains belonging to the same species. Namely, the strains identified as *R. mucilaginosa* WUT10 and WUT60, unlike WUT167, exhibited growth up to 30 °C. Similarly, for two *Sporobolomyces roseus* strains, the maximum growth temperature varied between 25 °C and 30 °C for WUT61 and WUT182, respectively.

Although many enzymes with industrial applications are produced using yeasts [28,29,30], new and safe sources of enzyme preparations are still being sought. Therefore, the ability of newly isolated strains to produce extracellular enzymes was also verified (Table 2). Several yeasts have been described as lipase producers, and among them species of the genus *Candida* are the most commercially used ones [31]. However, lipase-producing basidiomycetes species have also been isolated and characterized [2,20,32,33,34,35]. In accordance with recent reports, in this study most of the strains were able to synthesize lipases and gave positive results in the plate tests (Table 2 and Figure S1), w hereas, a proteolytic activity was only demonstrated for *Cystofilobasidium macerans* WUT145 (Figure S2). Till now only one paper has described protease production by yeast from the *Cystofilobasidium* genus [36], however, other cold-adapted yeasts belonging to basidiomycetes were previously characterized as potential protease producers [37,38]. Additionally, within the tested strains, four of them, *Sporobolomyces roseus* WUT61, *C. macerans* WUT145, *S. pararoseus* WUT159 and *S. roseus* WUT182, produced clear positive results for cellulolytic activity in the plate test (Figure S3). It is worthwhile noting that the authors are un aware of any works evaluating the potential of *Cystofilobasidium* or *Sporobolomyces* species for cellulases production, and little has been published about *R. glutinis* with cellulolytic ability [39].

The ability to utilize various carbon sources, especially combined with the extracellular hydrolytic enzymes production is desired in term of designing bioprocesses conducted in media based on agricultural waste (e.g., lignocellulosic biomass in form of straw), which is rich in polymers composed of sugars like glucose, xylose, galactose or cellobiose. Thus, the cellulolytic activity of the isolated yeasts was studied in more detail and presented below.

### 3.3. Synthesis of Cellulases by Selected Yeasts

**Figure 2:**
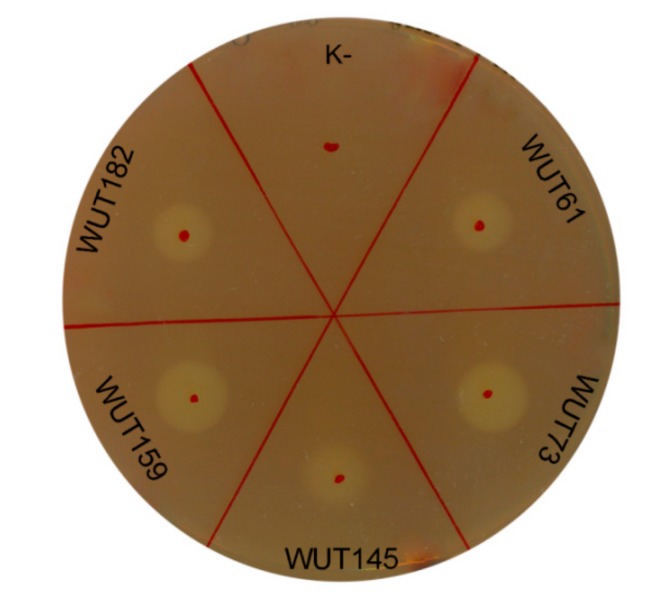
Cellulolytic activity determined by plate assay. Ten μL of cell-free supernatants from 4-days liquid cultures were spotted onto the plates containing 2% CMC. Plates were incubated overnight at 22°C and stained with Gram’s iodine solution after incubation. As a positive control, cellulolytic strain *Aureobasidium pullulans* WUT73 was used. “K-”—negative control (10 μL of pure medium).

Cellulases are the enzymes responsible for the hydrolysis of cellulose into sugars, and cellulose is the most abundant source of renewable energy on the planet. Therefore, cellulases are widely applied in various sectors, such as food, detergent, laundry, textile, baking and bio-fuels industries. However, the commercially available cellulolytic enzymes are mostly active at elevated temperature, about 40–50 °C, like one of the most popular enzyme coctail Cellic^®^ CTec2 and HTec2 [40]. One of the key requirements in these processes is to reduce the costs of heating, leading to a search for microorganisms, especially psychrotolerant ones, that secrete cellulases active at moderate temperatures. To date, very few studies have reported the production of cellulases by yeasts, mainly *Rhodotorula mucilaginosa* [41], *Aureobasidium pullulans* [42] or *Pseudozyma brasiliensis* [43]. Thus, the search for new cellulolytic yeast is of a great importance. From the preliminary screening described above, four strains (*S. roseus* WUT61, *C. macerans* WUT145, *S. pararoseus* WUT159 and *S. roseus* WUT182) with this trait were found (Table 2). They were then subjected to further analyses in terms of cellulolytic activity, by applying two assays – plate and liquid ones. Yeast cultures were grown for 4 days at 22°C in an YPDC medium containing 1% carboxymethylcellulose (CMC) to induce the production of cellulases. The cellular-free supernatants were then tested for cellulolytic activity. For all tested supernatants, light halos were observed in plate assay, which indicated the cellulose degradation (Figure 2).

Although the halo diameters were similar for each supernatant culture, the cellulolytic activities determined in liquid enzyme assay revealed significant differences (Table 3). The cellulolytic activity obtained for strain *S. pararoseus* WUT159, which exhibited the highest halo diameter (11.0 ± 0.0 mm) in the plate test, was the lowest and reached a rate of merely 0.30 ± 0.10 µmol/mg protein·min, while for *C. macerans* WUT145, with the smallest halo diameter (8.7 ± 1.4 mm) in plate test, a value over 20 times higher was obtained, 66.23 ± 0.15 µmol/mg protein·min. Several factors may have influenced the results obtained in plate test, which can be considered more qualitative rather than quantitative, as has also been discussed by others [44]. Consequently, tests carried out in liquid seem to be more credible. The WUT145 strain exhibited extraordinary cellulolytic activity, and, to the authors’ best knowledge, this is the first report which demonstrates the *C. macerans* strain with the above-mentioned characteristic. In addition, unlike for commercially available cellulases which are active at 40–50 °C, the strong cellulolytic activity of WUT145 strain was determined at 22 °C, which is highly desired by the industry. Also, for the *Sporobolomyces* genus strains, no reports on cellulolytic activity can be found. Thus, the results obtained here can be considered promising and it would be worthwhile to continue research on cellulolytic activity of selected strains, especially on *C. macerans* WUT145.

### 3.4. Preliminary Evaluation of Carotenoid Production

Based on the colour of the colony, 16 yeast strains were tested for carotenoid biosynthesis. The studies assume that the optimum temperature for the production will be 25 °C (maximum temp. growth observed for all tested strains in previous studies). Although the first eight strains (WUT10-WUT117) cultivated at 25 °C exhibited moderate growth (estimated biomass production (X) was between 4.5–7.4 g/L), the remaining eight strains (WUT128-WUT194) grew poorly under the tested conditions. Therefore, the temperature was reduced to 22 °C. This change resulted in a considerable improvement in yeast growth (calculated X was between 6–9.7 g/L) (Table 4). Together with the biomass measurements, at the end of the culture, residual glucose concentration was also estimated by HPLC. Glucose was only determined in the cultures of 3 strains: *Cystobasidium laryngis* WUT89, *Cystobasidium laryngis* WUT103 and *Cystobasidium psychroaquaticum* WUT117, the remaining strains utilized the dextrose present in medium. After a 4-day incubation, yeast biomass was used to determine the lipids and carotenoids content.

#### 3.4.1. First Test Group—Strains WUT10-WUT117

The total carotenoid content (C_C_), expressed as mg carotenoids/g biomass (Table 4), varied between the tested strains, even for those belonging to the same genera. Strains WUT10 and WUT60 identified as *R. mucilaginosa* exhibited a 2-fold difference in total carotenoid content (0.23 ± 0.02 *vs* 0.11 ± 0.01 mg carotenoids/g biomass, respectively). No direct correlation between the yeast growth and the carotenoid production was observed, yet there may be a connection between carotenoid production and glucose intake. The least amount of carotenoids was determined in the strain cultures where the glucose was not entirely consumed (0.05–0.09 mg carotenoids/g biomass). For strains that utilized all the glucose during the 4-day incubation, the carotenoid content was almost 3.6-times higher (0.16–0.32 mg carotenoids/g biomass). The highest production, 0.32 ± 0.04 mg carotenoids/g biomass, was estimated for *Cystobasidium sp.* WUT92 strain.

As demonstrated in this study, identifiable carotenoids also differ between tested strains. *R. mucilaginosa* is known from biosynthesizing β-carotene as the major carotenoid [4]. However, species of *R. mucilaginosa* producing other carotenoids in predominance can also be found, eg. torularhodin [45]. Two strains *R. mucilaginosa* isolated within this study synthesized mainly torulene and torularhodin; this trait may be strain- and medium-specific.

For *Cystobasidium* strains, *C. laryngis* WUT89, *C. laryngis* WUT103 and *C. psychroaquaticum* WUT117, γ-carotene and β-carotene are the main synthesized carotenoids. For the best yeast producer from this test group, *Cystobasidium sp.* WUT92, all four carotenoids, of almost the same amount, were detectable (Table 4). Interestingly, strains from the genus *Cystobasidium* are not yet well characterized, especially in terms of the production of carotenoids.

#### 3.4.2. Second Test Group—strains WUT128-WUT194

Considerably better results were obtained with the remaining eight strains when the growth temperature was lowered to 22 °C. At the end of the cultivation, the DCW calculated was in a range of 5.97–9.70 g/L (Table 4). Quite like the first test group, carotenoid production was not directly linked with the production of yeast biomass. The weakest carotenoid biosynthesis, under tested conditions, was demonstrated by the strain *C. macerans* WUT145, for which the total carotenoid content reached value of 0.06 ± 0.02 mg carotenoids/g biomass. Almost a 3.4-fold higher result was obtained for the strain *S. roseus* WUT182, where the total carotenoid content was 1.12 ± 0.01 mg carotenoids/g biomass (Table 4, Figure S4). The second best carotenoid producer was *R. graminis* WUT128 (0.93 ± 0.10 mg carotenoids/g biomass). Among strains from this group, the carotenoid distribution is more even than in the first test group. Torularhodin constituted the main carotenoid component in five out of eight strains; the second pigment in terms of production was torulene. β-Carotene and γ-carotene were produced in a minority for almost all tested strains (except in *R. graminis* WUT194 and *S. pararoseus* WUT159).

### 3.5. The Effect of Lower Temperatures on Carotenoid Synthesis in a Batch Cultures of Cystobasidium Species 

Little is known about the yeasts from the *Cystobasidium* genus; however, this group is extensively studied as of late. From the preliminary screening for carotenoid bio-production described above, newly isolated strains assigned to *Cystobasidium* sp. would also appear to be valuable producers of natural dyes. Therefore, it was decided to study them further in this respect.

The influence on carotenoids production has many factors, e.g., effect of the carbon to nitrogen (C/N) ratio, sources of nitrogen and carbon, mineral salts and incubation temperature. Because all newly isolated strains were well adapted to cooler temperatures, it was decided to first evaluate the influence of reduced temperature, between 20 °C and 15 °C, on carotenoids biosynthesis. After a 4-day incubation biomass production, the presence of residual glucose concentration, lipids, and carotenoid production were determined (Table 5). For two strains, *C. laryngis* WUT89 and *C. psychroaquaticum* WUT117, the carotenoid output was linearly related with the temperature; the highest yields, 0.73 ± 0.22 mg/L and 3.05 ± 0.45 mg/L respectively, were achieved at 15 °C. At the same time, the biomass yield coefficient (Y_XS_) was also the highest (0.60 g biomass/g substrate and 0.41 g biomass/g substrate for WUT89 and WUT117) and decreased with a rise in temperature (to 0.44 g biomass/g substrate and 0.33 g biomass/g substrate, respectively). When evaluating the carotenoid content, the same trend was identified for *C. psychroaquaticum* WUT117. No significant differences between the three tested temperature variants were observed for *C. laryngis* WUT89. It should be noted that the production of carotenoid pigments was not related with the biosynthesis of lipids. The highest amount was estimated at 20 °C (between 2–4 times higher than at 15 °C, and a staggering 10–16 times higher at 25 °C) for both strains, and under these conditions, the highest glucose intake was also determined.

In contrast, the WUT92 strain behaved differently. The best carotenoid production occurred at 25 °C, 2.62 ± 0.03 mg/L,0.32 mg/g biomass (Table 4), and was more than 2-times higher than at 15 °C, 0.92 ± 0.06 mg/L, 0.14 ± 0.00 mg/g biomass. When comparing these two variants, a near 2-fold difference was also determined in lipid biosynthesis, in favour of the higher temperature. Simultaneously, *Cystobasidium sp.* WUT92 metabolized all the glucose present in medium at 25 °C, and reached the highest biomass production but consuming only half the initial glucose amount at 15°C. Intensified carotenoid production, unlike for the other two strains, was consequently linked with the decrease in the biomass yield coefficient. Similarly to WUT89 and WUT177, biosynthesis of lipids was the highest at 20 °C, and for WUT92 was associated with the lowest carotenoid output (0.24 ± 0.02 mg/L).

The distribution of pigments produced varied among experiments. While at 25 °C, the main pigments biosynthesized were β-carotene, γ-carotene, torulene and torularhodin, lowering the temperature significantly affected the distribution of the carotenoids, in favor for γ-carotene, and in one case (in culture of WUT92 at 20 °C) trace amounts of torularhodin. The chromatographic analyses revealed that also other, yet unknown carotenoid pigments were synthesized. Their identification requires, however, more complex analyses, such as e.g., HPLC-MS/MS [46]. These compounds may correspond to xanthophylls or to pigments from the apocarotenoid group. They could also be new, unfamiliar structures. Nevertheless, the strains used in the study are the species which are not well documented. The metabolic pathways leading to carotenoid synthesis in these strains are also still to be fully understood. Investigating the probable structure of pigments produced at a reduced temperature will be challenging, but it is certainly an attractive goal for further research, and could result in determining new species with interesting traits.

### 3.6. The Impact of Glucose Concentration on Pigment Accumulation in R. mucilaginosa and C. macerans Strains

Most yeast strains produce a mixture of pigments, with a varied composition. The biosynthesis of only one carotenoid in predominance seems to be atypical. Three strains isolated within this study, *R. mucilaginosa* WUT10 and WUT60, and *C. macerans* WUT145, exhibited this untypical trait in the preliminary tests (Table 4). The regulation of carotenoid production can be obtained by enhancing different factors associated with fermentation conditions [47]. *Rhodotorula* strains cultivated in medium containing a higher C/N ratio generally showed positively affected carotenoid synthesis [48,49]; but this dependence is strain-specific. The above-mentioned strains were first cultivated in a medium containing 20 g/L glucose, and under these conditions, their growth was limited (at the end of the culture biomass production reached between 6.7–6.9 g/L). In connection, the amount of synthesized carotenoids was also rather poor; it did not exceed 1.5 mg/L. Therefore, in order to examine whether an increased glucose titer could influence both yeast growth and productivity, while maintaining a single-pigment bio-production; the glucose content was increased from 20 g/L to 30 and 40 g/L (Table 6).

This study revealed that initial glucose titer had a great impact on both the productivity and the ratio of produced pigments. Higher glucose titer led to increased formation of biomass; 10.75 ± 0.35 g/L (30 g/L) and 16.60 ± 0.70 g/L (40 g/L) *vs* 6.86 ± 0.24 g/L (20 g/L); 10.43 ± 0.55 g/L (30 g/L) and 13.37 ± 0.55 g/L (40 g/L) *vs* 6.91 ± 0.01 g/L (20 g/L), for *R. mucilaginosa* WUT10 and WUT60 strains respectively; while negatively affecting pigments production. For both strains we observed a fall of between 26% and 59% in the total carotenoid content, accompanied by a lower yield of torularhodin (main carotenoid and the only detected upon cultivation on lower glucose titer) and the production of an additional pigment, γ-carotene or β-carotene. This may indicate some changes in the metabolic pathways, since γ-carotene is a direct precursor of β-carotene, and indirect of torularhodin [50]. In the case of *C. macerans* WUT145, similar to the other two strains, a higher glucose content influenced the intensified biomass production (up to 2-fold increase) in connection with the definite enhancement of carotenoid biosynthesis; the maximum carotenoid content was obtained at 40 g/L glucose, 1.72 ± 0.08 mg/L (0.13 ± 0.01 mg/g biomass) which was almost a 3-fold increase when compared to the initial cultures. Although, in comparison to *C. macerans* strain tested by Marova *et al.*, 2017 [48], which produced 1.98 mg carotenoids/g DCW on animal fat wastes, *C. macerans* WUT145 produced significantly less carotenoids; yet this strain has the potential to enhance its production, even on media containing alternative carbon sources deriving from wastes from various industries, thanks to its ability to secrete diverse extracellular enzymes. The productivity of individual pigments employing different media was also changed. The predominance of torulene was replaced by a mixture of torulene, torularhodin and γ-carotene. *C. macerans* WUT145 biosynthesize also small amounts of unidentified carotenoids under tested conditions.

## 4. Conclusions

The need for natural carotenoids has triggered research to examine commercially viable processes for their cost-effective production. The chemical synthesis of carotenoids is both expensive and challenging. Their extraction from raw materials is often limited by the source availability and the financial aspects. Therefore, an increasing interest in microbial sources for carotenoids has been observed. To date, in the literature, species of yeast strains, such as *Phaffia rhodozyma*, *Rhodotorula* spp., are described in detail. However, little is known about other non-conventional yeasts.

In this study, sixteen red-pink pigmented yeast strains were isolated and were classified into five genera: *Rhodotorula, Cystobasidium, Sporobolomyces, Sporidiobolus* and *Cystofilobasidium.* All of the newly selected yeasts were able to synthesize carotenoid pigments under tested conditions. In the preliminary tests, the highest carotenoid accumulation showed strains *Sporobolomyces roseus* WUT182, which was able to produce 1.12 ± 0.01 mg pigments/g biomass and *Rhodotorula graminis* WUT128, 0.93 ± 0.10 mg pigments/g biomass, but these strains differ in the distribution of carotenoids. It is worth emphasizing that, amongst the newly isolated strains, we found ones from the *Cystobasidium* cluster that are still poorly recognized in this respect, and therefore, the results obtained during this work are extremely valuable data. Interestingly, all the strains, although not collected from polar regions, are psychrophilic (optimal growth temperatures between 4°C and 22°C). For two strains *C. laryngis* WUT89 and *C. psychroaquaticum* WUT117 reducing the growth temperature resulted in a 10-fold increase in carotenoid synthesis. Further research on the optimization of culture conditions may probably bring subsequent increase in productivity.

As expected, most of isolated yeasts biosynthesized extracellular lipases. However, more interestingly, within the tested group, four cellulolytic strains (*S. roseus* WUT61, *C. macerans* WUT145, *S. pararoseus* WUT159 and *S. roseus* WUT182) and one proteolytic (*C. macerans* WUT145) were also revealed. Strains exhibiting the ability to secrete enzymes, and therefore utilize various organic wastes as substrates for their growth, can be considered alone or in mixed-culture fermentations for a development of carotenoids production on agro-industrial waste, e.g., lignocellulose or whey. However, additional experiments will be needed to design such processes to determine both the optimum growth temperature, the optimum cellulase/protease secretion temperature and the optimum carotenoid production temperature. Also the relation between this parameters will be key when developing technologies based on organic wastes. Furthermore, it would be interesting also to study in more detail enzymatic activity of selected yeast strains, as this group of enzymes has numerous applications; e.g., in the processing of fats and oils, as additives for detergents and degreasing agents, in food processing, in chemical and pharmaceutical synthesis, in the production of paper, the food and feed industry, agriculture and in the cosmetics industry [51,52].

## Figures and Tables

**Table 1:** Molecular identification of isolated yeast strains based on the sequences of ITS and D1/D2 regions in genomic DNA. *Based on BLASTn search and phylogenetic analysis. **Containing partial 18S rRNA gene, ITS1, 5.8S rRNA gene, ITS2 and partial 26S rRNA gene. *** Sequences were submitted to the NCBI GenBank database and are publicly available.

Strain	Source	Identification *	Sequence Length of Genomic DNA (bp) ** / GenBank Accession Number ***
WUT10	Fermented milk, Antalya, Turkey	*Rhodotorula mucilaginosa*	1120 / MN006686
WUT57	Wild strawberry shrub, Toulouse, France	*Rhodotorula graminis*	1111 / MN006688
WUT60	Pepper, Warsaw, Poland	*Rhodotorula mucilaginosa*	1112 / MN006694
WUT61	Beech tree, Barania Mountain, Poland	*Sporobolomyces roseus*	1110 / MN006697
WUT89	Birch bark, Słowiński National Park, Poland	*Cystobasidium laryngis*	1072 / MN006700
WUT92	Birch bark, Słowiński National Park, Poland	*Cystobasidium sp.*	968 / MN006698
WUT103	Birch bark, Warsaw, Poland	*Cystobasidium laryngis*	1103 / MN006701
WUT117	Mirabelle, Warsaw, Poland	*Cystobasidium psychroaquaticum*	1101 / MN006705
WUT128	Sow-thistle, Turku, Finland	*Rhodotorula graminis*	1018 / MN006772
WUT145	Tree leaf, Cork, Ireland	*Cystofilobasidium macerans*	1156 / MN006771
WUT147	Red grapes, Warsaw, Poland	*Rhodotorula graminis*	1005 / MN006773
WUT159	Apple, Warsaw, Poland	*Sporidiobolus pararoseus*	1092 / MN006774
WUT165	Apple, Warsaw, Poland	*Rhodotorula graminis*	1097 / MN006776
WUT167	Rowanberry, Warsaw, Poland	*Rhodotorula mucilaginosa*	1105 / MN006818
WUT182	Quince, Riga, Latvia	*Sporobolomyces roseus*	1095 / MN006819
WUT194	Grapes, Warsaw, Poland	*Rhodotorula graminis*	1107 / MN006820

**Table 2:** Physiological and biochemical characteristics of isolated yeast strains. YNB—yeast nitrogen base, GLU—glucose, XYL—Xylose, GAL—Galactose, LAC—lactose, SAC—saccharose, GLY—glycerol, EtOH—ethanol, CEL—cellobiose, MAL—maltose. ^a^ As a positive result, discoloration of the medium around colonies is observed. No changes in medium colour indicate negative result. ^b,c^ Positive result: Appearance of transparent zones around the colonies. No changes indicate negative result. “-” no growth/negative result, “+” growth/positive result, “w” weak growth/activity.

Strain Number in WUT Collection																
	10	57	60	61	89	92	103	117	128	145	147	159	165	167	182	194
**Growth (assimilation) on carbon compounds**																
**YNB + GLU**	+	+	+	+	+	+	+	+	+	+	+	+	+	+	+	+
**YNB + XYL**	+	w	w	−	+	+	+	+	+	w	w	−	−	w	−	−
**YNB + GAL**	+	w	+	−	−	+	−	−	+	−	+	w	+	+	−	w
**YNB + LAC**	−	−	−	−	−	−	−	−	−	−	−	−	−	−	−	−
**YNB + SAC**	+	+	+	+	+	+	+	+	+	−	+	+	+	+	+	+
**YNB + GLY**	+	w	−	+	+	+	+	+	+	w	w	w	−	w	−	w
**YNB + EtOH**	−	−	−	w	+	−	w	w	+	w	w	−	−	w	−	w
**YNB + CEL**	−	+	−	w	w	+	+	+	+	+	+	+	+	+	w	w
**YNB + MAL**	+	+	+	+	−	−	−	−	+	+	+	+	+	+	+	−
**Range of temperature growth (YPD medium, 2–3 days)**																
**[°C]**	4–30	4–30	4–30	4–25	4–25	4–25	4–25	4–25	4–30	4–25	4–30	4–30	4–25	4–37	4–30	4–30
**Extracellular enzymes production**																
**Lipase activity ^a^**	+	+	+	+	w	w	w	w	+	+	+	−	+	+	w	+
**Protease activity ^b^**	−	−	−	−	−	−	−	−	−	+	−	−	−	−	−	−
**Cellulase activity ^c^**	−	−	−	+	−	−	−	−	−	+	−	+	−	−	+	−

**Table 3:** Cellulolytic activity of four selected strains expressed as halo diameter in a plate assay and CMC activity in liquid enzyme assay. * Measured in duplicate from two biological repetitions (*n* = 4).

Strain	Halo Diameter [mm]	CMC Activity* [µmol/mg Protein*min]
**WUT61**	9.7 ± 0.5	0.65 ± 0.21
**WUT145**	8.7 ± 1.4	66.23 ± 0.15
**WUT159**	11.0 ± 0.0	0.30 ± 0.10
**WUT182**	10.0 ± 1.4	3.54 ± 0.76
**WUT73 (K+)**	11.3 ± 0.9	1.55 ± 0.09

**Table 4:** Four day carotenoid production cultures of isolated yeast strains at 25 °C and 22 °C. X—biomass production, L—lipidic extract, C_C_—total carotenoid content, Y_C_—carotenoid output, nd—not detected, a—the average cannot be calculated; *—bolded names denote a predominant dye. **^#^** highest values.

Strain	X [g/L]	Residual Glucose [g/L]	L [mg]	C_C_ [mg/g Biomass]	Y_C_ [mg/L]	Detected Carotenoids *
**First test group (at 25 °C)**						
**WUT10**	6.86 ± 0.24	nd	1.85 ± 0.76	0.23 ± 0.02	1.49 ± 0.10	**torularhodin**
**WUT57**	4.55 ± 0.22	nd	a	0.25 ± 0.02	1.10 ± 0.10	**torularhodin**, β-carotene, γ-carotene
**WUT60**	6.91 ± 0.10	nd	8.95 ± 0.99	0.11 ± 0.01	0.66 ± 0.10	**torularhodin**
**WUT61**	7.37 ± 0.21	nd	a	0.16 ± 0.00	1.18 ± 0.00	**torularhodin**, β-carotene, γ-carotene, torulene
**WUT89**	4.65 ± 0.29	9.09 ± 0.15	8.30 ± 0.42	0.09 ± 0.01	0.41 ± 0.09	**β-carotene, γ-carotene**, torulene, torularhodin
**WUT92**	7.55 ± 0.25	nd	22.30 ± 1.56	0.32 ± 0.04	2.62 ± 0.03	**torularhodin, torulene**, **β-carotene**, γ-carotene
**WUT103**	4.51 ± 0.17	9.25 ± 0.67	5.10 ± 0.72	0.05 ± 0.01	0.25 ± 0.05	**β-carotene, γ-carotene**, torulene, torularhodin
**WUT117**	4.42 ± 0.06	6.50 ± 0.51	8.00 ± 0.57	0.06 ± 0.01	0.31 ± 0.04	**β-carotene, γ-carotene, torularhodin**, torulene
**Second test group (at 22 °C)**						
**WUT128**	7.10 ± 0.26	nd	25.70 ± 5.52	0.93 ± 0.10	6.61 ± 0.92 ^#^	**torularhodin**, β-carotene, γ-carotene, torulene
**WUT145**	6.70 ± 0.90	nd	7.40 ± 0.42	0.06 ± 0.012	0.62 ± 0.02	**torulene**
**WUT147**	9.70 ± 1.27	nd	13.85 ± 3.61	0.60 ± 0.06	6.00 ± 0.52	**torularhodin**, β-carotene, γ-carotene, torulene
**WUT159**	7.17 ± 0.23	nd	13.45 ± 3.89	0.75 ± 0.06	5.35 ± 0.51	**torularhodin**, β-carotene, γ-carotene, torulene
**WUT165**	9.07 ± 0.75	nd	4.20 ± 0.28	0.35 ± 0.05	3.06 ± 0.09	**torularhodin**, β-carotene, torulene
**WUT167**	8.67 ± 0.32	nd	8.95 ± 1.34	0.34 ± 0.03	3.09 ± 0.21	**torularhodin**, β-carotene, torulene
**WUT182**	9.23 ± 0.21	nd	19.70 ± 0.28	1.12 ± 0.01	10.33 ± 0.24 ^#^	**torulene**, torularhodin, β-carotene, γ-carotene
**WUT194**	5.97 ± 0.15	nd	7.70 ± 0.99	0.35 ± 0.05	3.06 ± 0.09	**torularhodin**, β-carotene, γ-carotene

**Table 5:** The effect of temperature on carotenoid production in three *Cystobasidium* species. X—Biomass production, L—lipidic extract, C_C_—total carotenoid content, Y_C_—carotenoid output, nd—not detected. **^#^** highest values.

Strain	X [g/L]	Residual Glucose [g/L]	L [mg]	C_C_ [mg/g Biomass]	Y_C_ [mg/L]	Detected Carotenoids
**15 °C**						
**WUT89**	7.58 ± 0.28	7.45 ± 0.24	34.00 ± 6.82	0.10 ± 0.03	0.73 ± 0.22 ^#^	γ-carotene
**WUT92**	6.48 ± 0.38	9.95 ± 0.18	12.35 ± 1.71	0.14 ± 0.00	0.92 ± 0.06	γ-carotene
**WUT117**	5.13 ± 0.32	7.42 ± 0.13	45.40 ± 3.48	0.54 ± 0.08	3.05 ± 0.45 ^#^	γ-carotene
**20 °C**						
**WUT89**	7.15 ± 0.82	4.83 ± 0.24	137.70 ± 12.59	0.10 ± 0.02	0.58 ± 0.14	γ-carotene
**WUT92**	6.93 ± 0.42	6.85 ± 0.27	87.50 ± 5.82	0.15 ± 0.04	0.24 ± 0.02	γ-carotene, torularhodin
**WUT117**	6.43 ± 0.30	3.78 ± 0.07	85.35 ± 4.19	0.14 ± 0.09	1.05 ± 0.30	γ-carotene

**Table 6:** Influence of increased glucose concentration on carotenoid production and pigment distribution by *R. mucilaginosa* and *C. macerans* strains. **^#^** highest values.

Strain	X [g/L]	C_C_ [mg/g Biomass]	Y_C_ [mg/L]	Carotenoid Distribution [mg/L]			
				β-Carotene	γ-Carotene	Torulene	Torularhodin
**30 g/L glucose**							
**10**	10.75 ± 0.35 **^#^**	0.04 ± 0.00	0.38 ± 0.05	0.06 ± 0.01	0	0	0.32 ± 0.04
**60**	10.43 ± 0.55	0.02 ± 0.00	0.17 ± 0.03	0	0.04 ± 0.01	0	0.15 ± 0.02
**145**	10.43 ± 1.27	0.08 ± 0.03	0.95 ± 0.06	0	0.33 ± 0.02	0.24 ± 0.04	0.13 ± 0.01
**40 g/L glucose**							
**10**	16.60 ± 0.70	0.04 ± 0.01	0.64 ± 0.11	0	0.07 ± 0.01	0	0.64 ± 0.04
**60**	13.37 ± 0.55	0.04 ± 0.01	0.39 ± 0.06	0	0.05 ± 0.01	0	0.34 ± 0.05
**145**	13.37 ± 0.55	0.13 ± 0.01	1.72 ± 0.08 ^#^	0	0.51 ± 0.02	0.56 ± 0.04	0.23 ± 0.03

## Supplementary Materials

The following are available online at https://www.mdpi.com/2076-2607/7/12/653/s1, Figure S1. Lipase activity plate tests after (a) 24 h and (b) 48 h incubation. The selected yeast cultures were grown at 22°C on plates containing Spirit Blue agar (Becton Dickinson, New Jersey, USA) with 20% (v/v) emulsion olive oil/Tween80. The expression of lipolytic enzymes was confirmed by a zone of clearance around the colony. Activity was determined based on results after 24 h incubation (Table 2 in main manuscript). Figure S2. Protease activity plate tests. The protease activity was tested on nutrient agar supplemented with 2% starch. The selected yeast cultures were grown at 22 °C for 3 days. The expression of proteases was confirmed by a zone of clearance around the colony. Figure S3. Cellulase activity plate tests. Cellulolytic activity was tested on yeast nitrogen base without aminoacids and with ammonium sulfate with 2% CMC. The selected yeast cultures were grown at 22 °C for 3 days. The positive expression of cellulase activity was indicated by a zone of clearance around the colony, which was visualized more clearly after pouring Lugol′s iodine onto the plate and incubating plates for 5 min. Figure S4. Typical HPLC-chromatogram of carotenoid extract obtained from batch culture of WUT165 recorded at 450 nm using diode array detector. Each carotenoid pigment was identified at the wavelength of maximum peak absorbance: torularhodin (λmax 507 nm; retention time 11.5 min, torulene (λmax 484 nm; retention time 13.4 min), β-carotene (λmax 450 nm; retention time ~15.0 min). Table S1. Cell size measurements. Cell sizes were calculated from at least 100 cells grown on SAB agar plates for 3 days at 22 °C. Microscopic observations were conducted through a 100× objective lens under brightfield (Levenhuk D870T with ToupView software).
