# Supplementary material for: Carotenoid-Producing Yeasts: Identification and Characteristics of Environmental Isolates with a Valuable Extracellular Enzymatic Activity
Source: Microorganisms. 2019 Dec 4;7(12):653. doi: 10.3390/microorganisms7120653 (PMC6956281; doi:10.3390/microorganisms7120653)
Supplement: Supplementary file 1 [file microorganisms-07-00653-s001.zip › Supplementary Table S1.docx]

| **Yeast strain** | **Cell size [µm]***  **(length x width)** |
| --- | --- |
| *Rhodotorula mucilaginosa* WUT10 | 4.96±0.71 x 3.77±0.47 |
| *Rhodotorula graminis* WUT57 | 6.03±0.74 x 3.23± 0.37 |
| *Rhodotorula mucilaginosa* WUT60 | 4.43±0.59 x 3.45±0.52 |
| *Sporobolomyces roseus* WUT61 | 6.71±1.38 x 3.94±0.64 |
| *Cystobasidium laryngis* WUT89 | 7.03±0.15 x 4.35±0.39 |
| *Cystobasidium sp.*WUT92 | 5.18±0.64 x 3.73±0.35 |
| *Cystobasidium laryngis* WUT103 | 5.09±0.70 x 3.20±0.50 |
| *Cystobasidium psychroaquaticum* WUT117 | 6.63±0.32 x 4.06±0.42 |
| *Rhodotorula graminis* WUT128 | 5.28±0.85 x 3.54±0.55 |
| *Cystofilobasidium macerans* WUT145 | 5.79±0.99 x 3.32±0.71 |
| *Rhodotorula graminis* WUT147 | 5.68±0.84 x 3.47±0.56 |
| *Sporidiobolus pararoseus* WUT159 | 8.47±1.64 x 4.01±0.80 |
| *Rhodotorula graminis* WUT165 | 6.26±0.88 x 3.70±0.66 |
| *Rhodotorula mucilaginosa* WUT167 | 5.66±0.81 x 4.61±0.72 |
| *Sporobolomyces roseus* WUT182 | 6.20±1.24 x 4.27±0.79 |
| *Rhodotorula graminis* WUT194 | 5.64±0.81 x 3.78±0.60 |

**Table S1. Cell size measurements.** Cell sizes were calculated from at least 100 cells grown on SAB agar plates for 3 days at 22°C. Microscopic observations were conducted through a 100× objective lens under brightfield (Levenhuk D870T with ToupView software).
