# Supplementary material for: Carotenoid-Producing Yeasts: Identification and Characteristics of Environmental Isolates with a Valuable Extracellular Enzymatic Activity
Source: Microorganisms. 2019 Dec 4;7(12):653. doi: 10.3390/microorganisms7120653 (PMC6956281; doi:10.3390/microorganisms7120653)
Supplement: Supplementary file 1 [file microorganisms-07-00653-s001.zip › Suplementary - Calculations.docx]

**Calculations**

$Dry cell weight \left( DCW \right)=\frac{mass of the empty tube}{mass of the tube with dried biomass}[g]$

$Biomass production \left( X \right)=\frac{DCW}{litre of culture} \left[ \frac{g}{L} \right]$

$$Biomass yield coefficient (Y_{XS})= \frac{\Delta X}{\Delta S} [\frac{g biomass}{g substrate}]$$

$Lipidic extract \left( L \right)=flask after chloroform evaporation mass-empty flask mass[mg]$

$Total carotenoid content \left( C_{C} \right)=\frac{{carotenoid mass}^{*}}{DCW used in extraction} [\frac{mg}{g biomass}]$

$Carotenoid output \left( Y_{C} \right)=C_{C}\cdot X [\frac{mg}{L}]$

*Carotenoid mass evaluated according to HPLC analyses
