# Supplementary figures and images for: Carotenoid-Producing Yeasts: Identification and Characteristics of Environmental Isolates with a Valuable Extracellular Enzymatic Activity
Source: Microorganisms. 2019 Dec 4;7(12):653. doi: 10.3390/microorganisms7120653 (PMC6956281; doi:10.3390/microorganisms7120653)

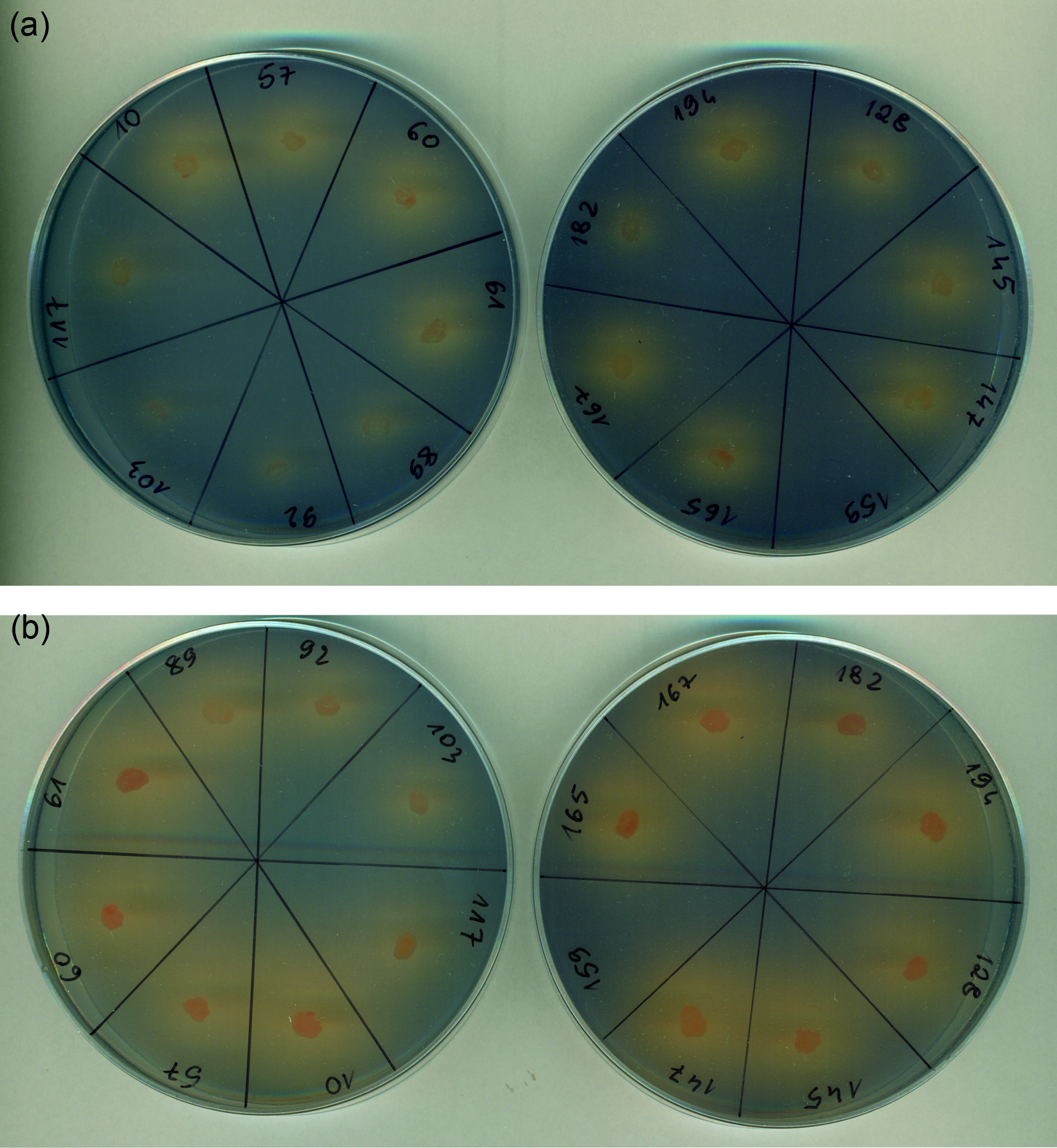

Supplement: Supplementary file 1 [file microorganisms-07-00653-s001.zip › Figure S1.tif]

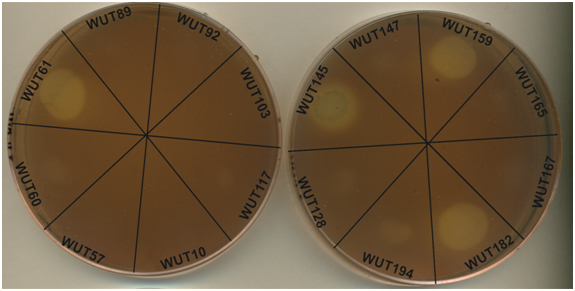

Supplement: Supplementary file 1 [file microorganisms-07-00653-s001.zip › Figure S3.tif]

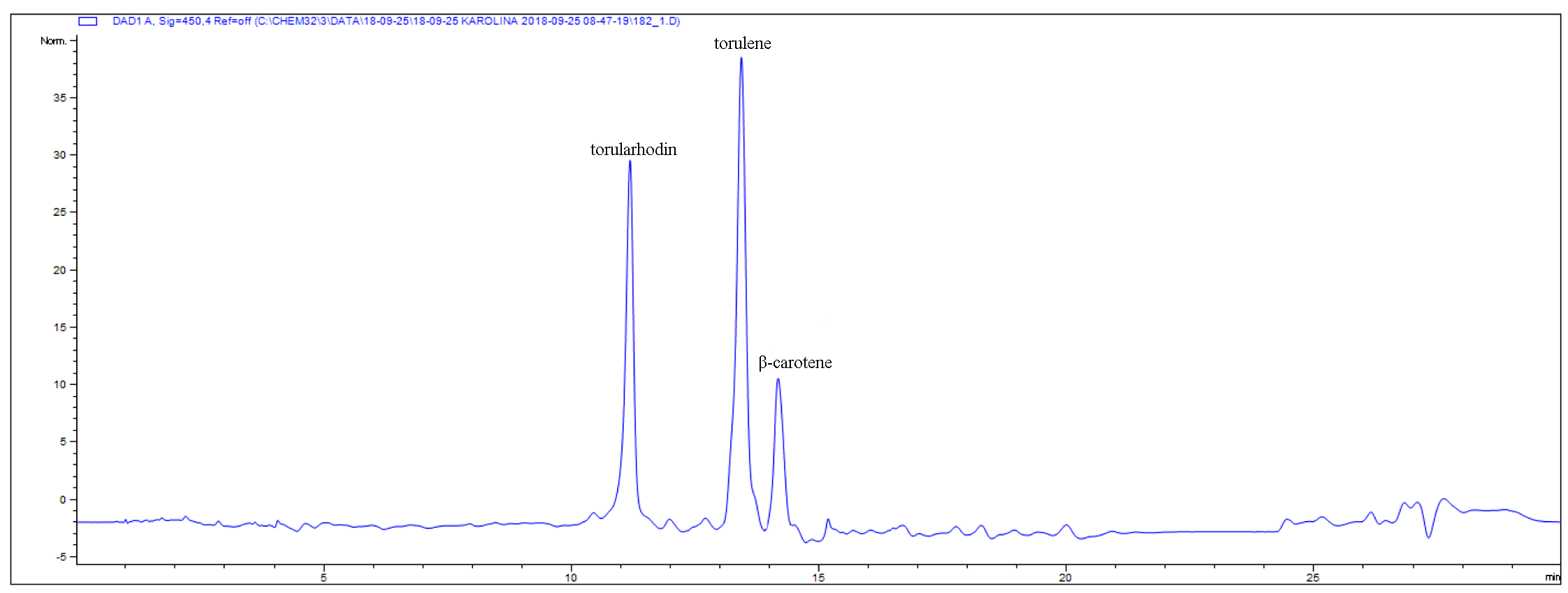

Supplement: Supplementary file 1 [file microorganisms-07-00653-s001.zip › Figure S4.tif]
